# Lenvatinib combined with nivolumab in advanced hepatocellular carcinoma-real-world experience

**DOI:** 10.1007/s10637-022-01248-0

**Published:** 2022-04-28

**Authors:** Wen-Chi Wu, Tzu-Yuan Lin, Ming‑Huang Chen, Yi‑Ping Hung, Chien-An Liu, Rheun‑Chuan Lee, Yi‑Hsiang Huang, Yee Chao, San-Chi Chen

**Affiliations:** 1 Division of Medical Oncology, Center of Immuno-Oncology, Department of Oncology, Taipei Veterans General Hospital, No. 201, Sec. 2, Shipai Road, Taipei, 11217 Taiwan; 2 Division of Hematology, Department of Medicine, Taipei Veterans General Hospital, Taipei, 11217 Taiwan; 3 Faculty of Medicine, National Yang Ming Chiao Tung University, Taipei, 11217 Taiwan; 4 Institute of Clinical Medicine, National Yang Ming Chiao Tung University, Taipei, 11217 Taiwan; 5 Department of Radiology, Taipei Veterans General Hospital, Taipei, 11217 Taiwan; 6 Division of Gastroenterology and Hepatology, Department of Medicine, Taipei Veterans General Hospital, Taipei, 11217 Taiwan

**Keywords:** Lenvatinib, Nivolumab, Anti-PD-1, Hepatocellular carcinoma (HCC), Vascular endothelial growth factor (VEGF), Fibroblast growth factors receptors (FGFR)

## Abstract

Lenvatinib, a multi-tyrosine kinase inhibitor that inhibits vascular endothelial growth factor and fibroblast growth factor receptors pathway, activated the immune response in tumor microenvironment. However, the combination of lenvatinib and anti-PD-1 has been reported in early phase studies. Hence, this study aims to explore the efficacy and toxicity of lenvatinib combined with nivolumab in the real-world setting. Advanced HCC patients who underwent lenvatinib combined with nivolumab (L + N group) treatment at Taipei Veterans General Hospital (Taipei, Taiwan) were reviewed between January 2016 and December 2020. Treatment response and outcomes were collected and analyzed. A control group with lenvatinib (L group) was also included for comparison. Forty patients were included in L + N group and 47 in L group. The L + N group demonstrated a higher objective response rate than L group (45.0% vs. 23.4%, *p* = 0.03). The L + N group also achieved longer PFS (7.5 vs. 4.8 months, *p* = 0.05) and OS (22.9 vs. 10.3 months, *p* = 0.01) than L group. Patients with HBV infection and REFLECT criteria fit demonstrated a trend of better prognosis. The PFS for those with PR, SD and PD groups were 11.2, 6.4, and 2.2 months and OS were non-reached, 14.6 and 4.7 months, respectively. Portal vein thrombosis (HR 4.3, 95% C.I. 1.5–12.8) and AFP > 400 ng/mL (HR 3.3, 95% C.I. 1.1–9.3) were poor prognostic factors and nivolumab used remained a protective factor (HR 0.2, 95% C.I. 0.1–0.7). Dermatitis (35.0%), pruritis (27.5%), and hypothyroidism (27.5%) were the common toxicities. Few patients developed grade 3/4 toxicities, including dermatitis (15%), gastrointestinal bleeding (7.5%), hypertension (5.0%), pneumonitis (2.5%) and stomatitis (2.5%). This is the first real-world data reporting the promising efficacy and tolerable toxicities of lenvatinib combined with nivolumab in advanced HCC. Further randomized trials are prompted.

**Supplementary information:**

The online version contains supplementary material available at 10.1007/s10637-022-01248-0.

## Introduction

Hepatocellular carcinoma (HCC) is the fourth most common cause of cancer-related death worldwide and second most common in Taiwan [1, 2]. For patients with advanced HCC, the prognosis is poor. Sorafenib, a multiple kinase inhibitor, has been the only approved drug for a decade, but the efficacy is limited [3]. Recently, several drugs including lenvatinib, regorafenib, cabozantinib, and ramucirumab have been approved for the treatment of advanced HCC [4–7].Particularly, the combination of atezolizumab (anti-PD-L1) and bevacizumab (anti-VEGF monoclonal antibody)provided longer survival benefit than sorafenib and has become a new standard therapy in first-line treatment for advanced HCC [8]. However, little is known for the combination of multi-kinase inhibitor and anti-PD-1.

Lenvatinib is a multi-tyrosine kinase inhibitor that affects `1–4, platelet-derived growth factor receptor-alpha (PDGFRa), RET, and KIT [9]. By blocking these pathways, it reduces angiogenesis and suppresses tumor growth. Its potent inhibition of FGFR pathway is considered the primary mechanism for controlling liver cancer. In REFLECT study, lenvatinib demonstrated better response and longer PFS, and non-inferiority of survival than that of sorafenib [4].

Immune checkpoint inhibitors have been widely studied in various cancers. Nivolumab and pembrolizumab, two anti-PD-1 agents, become breakthrough therapies in the second-line treatment of advanced HCC [10, 11]. However, in a phase 3 clinical trial (CheckMate-459), nivolumab failed to prove its superiority to sorafenib in the first-line treatment [12]. Therefore, to explore a potential drug for combination becomes the future development for nivolumab.

At present, more and more evidence has shown that VEGF pathway inhibitors have immunomodulatory effects [13]. The combination of VEGF pathway inhibitors and anti-PD-L1 improves treatment efficacy and becomes standard treatment in HCC [8]. Many multi-kinase inhibitors that block VEGF pathway have been proved to have immunomodulatory effects, including lenvatinib [14]. Lenvatinib, a potent FGFR inhibitor, not only suppressed the progression of HCC, but also activated the immune response in tumor microenvironment [15]. Therefore, lenvatinib is potential for the combination of anti-PD-1. However, only a Phase 1 clinical trial showed the efficacy of lenvatinib combined with nivolumab, and no real-world data is reported to respond to the clinical trial [16]. Hence, the purpose of this study is to explore the clinical efficacy and side effects of lenvatinib combined with nivolumab in the real-world settings.

## Methods

### Patients and study design

Between January 2016 and December 2020, patients with advanced HCC who underwent lenvatinib combined nivolumab (L + N group) at Taipei Veterans General Hospital (Taipei, Taiwan) were retrospectively reviewed. Patients who have Child–Pugh Score C, aged less than 20-year-old, or those without effective assessment were excluded. A total of 40 patients were enrolled after screening. To compare the efficacy between combination therapy and lenvatinib monotherapy, additional 47 HCC patients underwent lenvatinib (L group) as 1^st^ line therapy with the same inclusion criteria were enrolled. Lenvatinib was given 8 or 12 mg based on body weight and nivolumab was given 1–3 mg/kg every 2 weeks. Patient characteristics, such as age, gender, etiologies, liver function, tumor stage, tumor marker, and previous local therapy and systemic therapy history were collected and analyzed. Child–Pugh score and ALBI grade were used to describe liver function, and Barcelona clinic liver cancer (BCLC) staging system to cancer staging. The diagnosis of HCC was defined as histological confirmation or clinical interpretation based on the American Association for the Study of Liver Diseases (AASLD) criteria [17].

### Outcome assessment

Treatment response was assessed by computed tomography scans or magnetic resonance imaging every 2–3 months. The treatment response including complete response (CR), partial response (PR), stable disease (SD), and progressive disease (PD) were reviewed by two independent specialists according to RECIST and mRECIST criteria (19). Treatment adverse events were graded according to the common terminology criteria for adverse events (CTCAE) version 5.0. Overall survival (OS) was defined as the period from the beginning of treatment to death; progression-free survival (PFS) from the beginning of the treatment to disease progression or death.

### Statistical analysis

For comparison of continuous variables, the student's t-test was used. For categorical variables between groups, the Chi-square test or Fisher's exact test was performed. We used logistic regression analysis to find the risk factors for death. In the univariate analysis, variables with *p* < 0.1 underwent multi-variates analysis using forward stepwise model. A *p* < 0.05 was considered as an independent prognostic factor. The Kaplan–Meier curves were compared by the Log-rank test. A *p* value < 0.05 was defined as a statistically significant difference. All the statistical analyses were performed by IBM® SPSS®, version 21.0 (IBM Corp. Released 2012. IBM SPSS Statistics for Windows, Version 21.0. Armonk, NY: IBM Corp.).

## Results

### Patient characteristics

Between January 2016 to December 2020, 40 HCC patients received lenvatinib in combination with nivolumab in our center were included. Among them, twenty-nine patients were male. The mean age was 58.5 ± 13.8; 77.5% (29/40) had HBV, 10.0% (4/40) had HCV, and 37.5% (15/40) had alcoholism history. Child–Pugh score A accounted for 62.5% of patients and Child–Pugh score B for 37.5%. Fifty percent of patients were diagnosed to have portal vein thrombosis and 25% already had distant metastasis. Regard to previous sorafenib treatment, 50% of patients has been experienced and 50% were sorafenib naïve. Additionally, 47 HCC patients with lenvatinib monotherapy were enrolled. Lenvatinib group had similar liver function and tumor stage as combination group, but older age and less HBV infection. Notably, all the patients in lenvatinib group were first-line treatment. The subsequent treatment was given in 84.4% (27/32) and 77.8% (21/27) in L + P and L group, respectively (*p* = 0.52). The detailed demographic and clinical characteristics were shown in Table 1.

**Table 1:** Clinical characteristics

**Characteristic**	**Lenvatinib + Nivolumab (n = 40)**		**Lenvatinib (n = 47)**		***p* value**
Age (year, mean ± SD)	58.5 ± 13.8		70.6 ± 13.3		< 0.01
Male	29	72.5.%	32	68.1%	0.65
Etiologies					
HBV	31	77.5%	26	55.3%	0.03
HCV	4	10.0%	11	23.4%	0.10
Alcohol	15	37.5%	12	26.7%	0.28
Child–Pugh score class					0.23
A	25	62.5%	35	74.5%	
B	15	37.5%	12	25.5%	
ALBI grade					0.40
Grade 1	12	30.0%	18	38.3%	
Grade 2	21	52.5%	25	53.2%	
Grade 3	7	17.5%	4	8.5%	
BCLC stage					0.69
B	17	42.5%	22	46.8%	
C	23	57.5%	25	53.2%	
PVT	20	50.0%	14	29.8%	0.05
Metastasis	10	25.0%	17	36.2%	0.26
AFP > 400 (ng/mL)	10	25.0%	18	38.3%	0.07
Previous drugs					
Sorafenib	20	50.0%	0	0%	< 0.01
Regorafenib	2	5.0%	0	0%	0.21
Cabozatinib	1	5.0%	0	0%	0.46
Line of treatment					< 0.01
1^st^ line	17	42.5%	47	100%	
2^nd^ line	16	40.0%	0	0%	
≧3^rd^ line	7	17.5%	0	0%	
Previous local therapy					
Surgery	10	25.0%	15	31.9%	0.48
RFA	6	15.0%	17	36.2%	0.03
TACE	23	57.5%	26	55.3%	0.84

*ALBI grade* albumin-bilirubin grade, *PVT* portal vein thrombosis, *AFP* alpha- fetoprotein, *RFA* radiofrequency ablation, *TACE* transarterial chemoembolization

### Treatment response

**Fig. 1:**
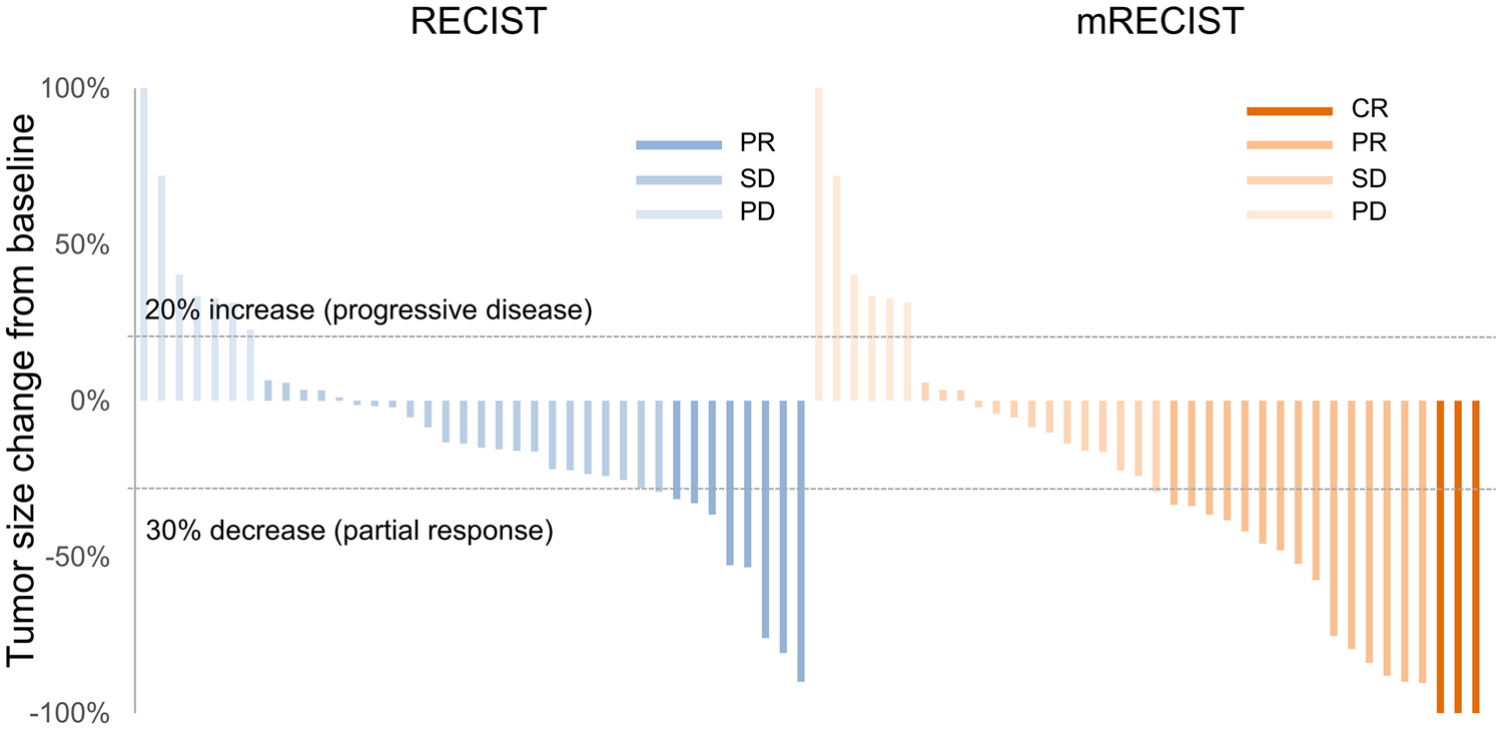
Maximum Change from Baseline in the Sum of Longest Diameters Lenvatinib plus nivolumab demonstrated remarkable tumor shrinkage and disease control by RECIST (left) and mRECIST criteria (right). PD was defined as 20% increase in tumor size, while partial response had a 30% decrease. (CR, complete response; PR, partial response; SD, stable disease; PD, progressive disease)

In the L + N group, the ORR was 45.0% and DCR 82.5% by mRECIST, and 20.0%, 82.5% by RECIST 1.1 (Fig. 1). Notably, three patient (7.5%) were defined as CR with mRECIST (one of the cases was shown in supplemental Fig. 2). Compared with the L group, the L + N group had a higher objective response rate (45.0% vs. 23.4%, *p* = 0.03). In the L + N group, patients with sorafenib naïve, HBV and HCV infection achieved numerically higher response rate without statistically significant (Table 2).

**Fig. 2:**
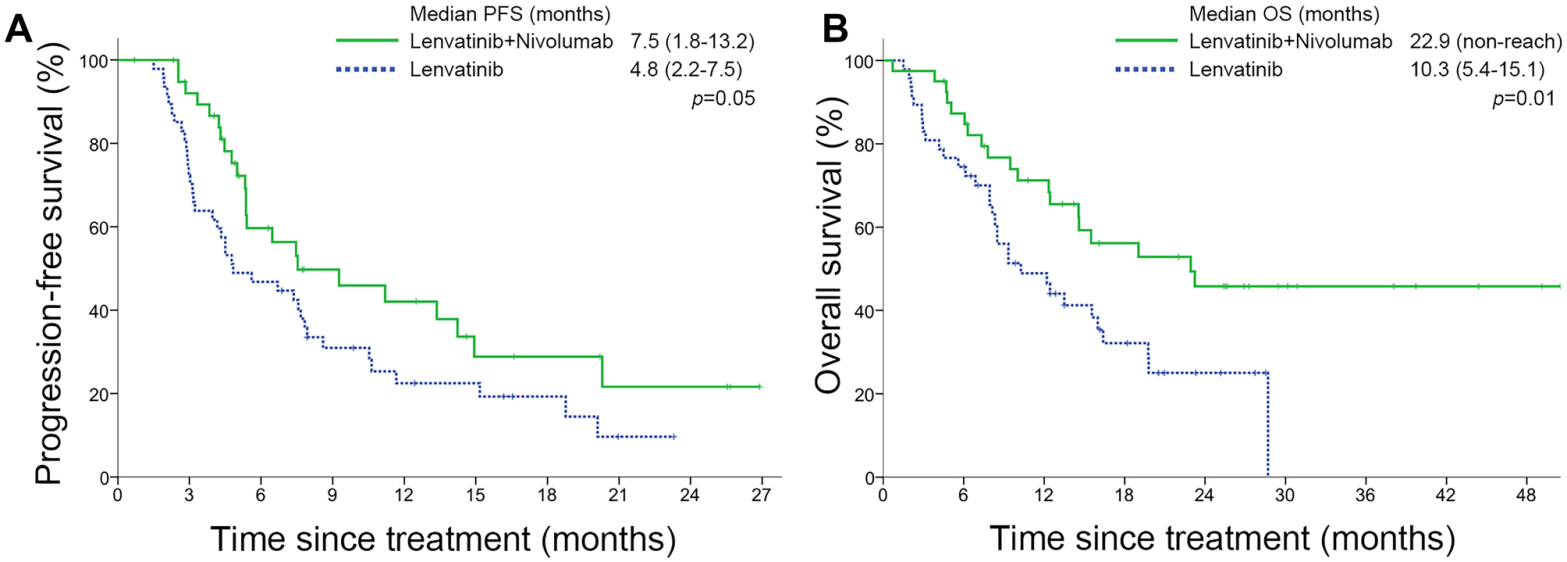
Kaplan–Meier curves for **A** progression-free survival and **B** overall survival stratified by treatment

**Table 2:** Treatment response by mRECIST criteria

**All patients**								
	CR	PR	SD	PD	ORR	*pv*	DCR	*pv*
Lenvatinib + nivolumab	7.5%	37.5%	37.5%	17.5%	45.0%	**0.03**	83.5%	0.50
Lenvatinib	6.4%	17.0%	28.7%	23.4%	23.4%		76.6%	
**Lenvatinib + nivolumab group**								
	CR	PR	SD	PD	ORR	*pv*	DCR	*pv*
Sorafenib								
Naïve	15.0%	40.0%	30.0%	15.0%	55.0%	0.20	85.0%	0.68
Experienced	0%	35.0%	45.0%	20.0%	35.0%		80.0%	
HBV								
Positive	6.5%	41.9%	35.5%	16.1%	48.4%	0.42	83.9%	0.65
Negative	11.1%	22.2%	44.4%	22.2%	33.3%		77.8%	
HCV								
Positive	0.0%	50.0%	25.0%	25.0%	50.0%	0.83	75.0%	0.68
Negative	8.3%	36.1%	38.9%	16.7%	44.4%		82.5%	
REFLECT criteria								
Fit	5.3%	42.1%	47.4%	5.3%	47.4%	0.78	94.7%	0.10
Unfit	9.5%	33.3%	28.6%	28.6%	42.9%		71.4%	

*CR* complete response, *PR* partial response, *SD* stable disease, *PD* progressive disease, *ORR* objective response rate, *DCR* disease-control rate, *pv p*-value

### Progression-free survival and overall survival

The L + N group achieved longer PFS (7.5 vs. 4.8 months, *p* = 0.05) and OS (22.9 vs. 10.3 months, *p* = 0.01) than L group (Fig. 2). Notably, the survival curve of L + N group presented with a long tail. The median follow-up time of all patients was 12.3 months (6.2–21.0).

**Fig. 3:**
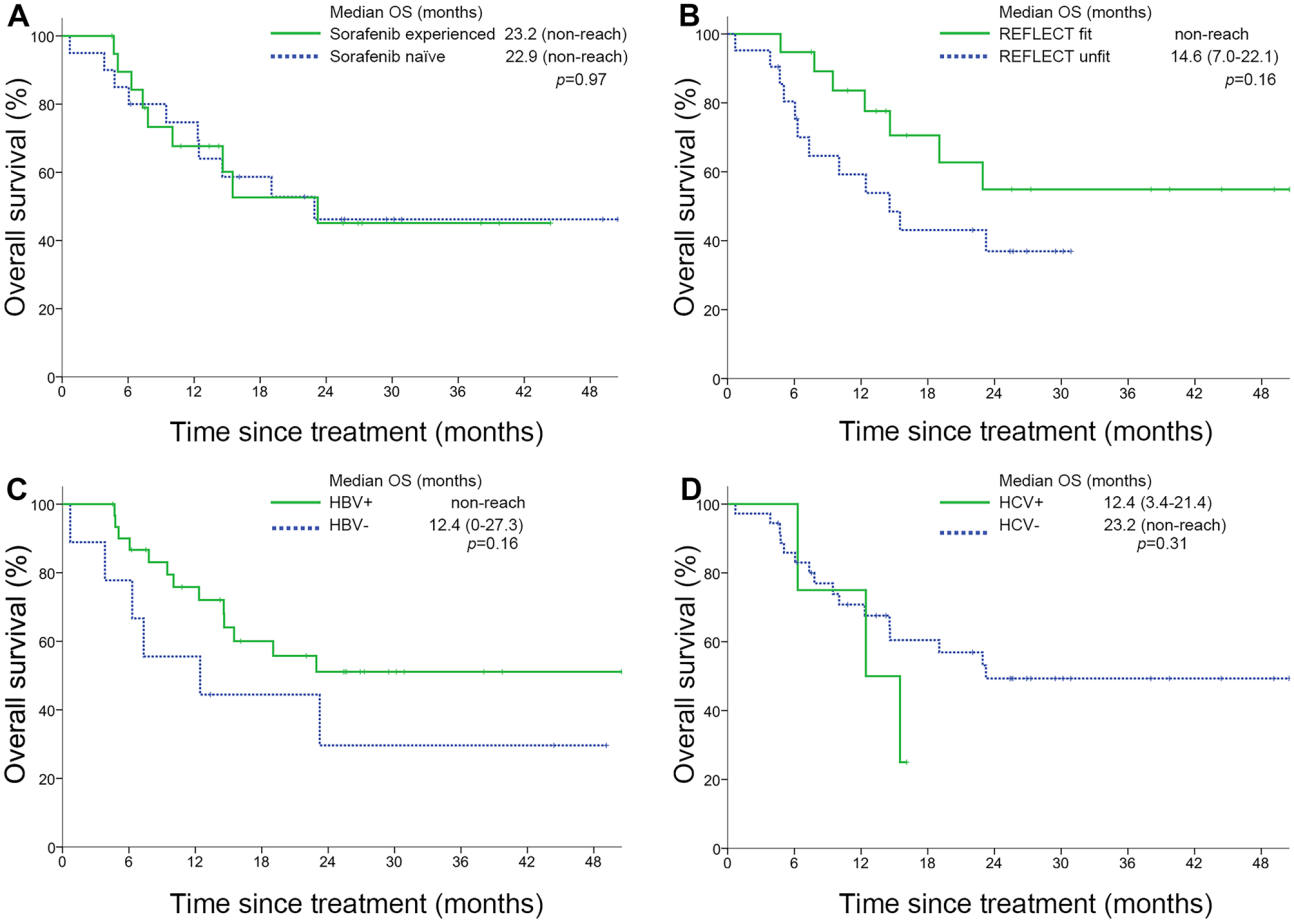
Kaplan–Meier curves for overall survival stratified by **A** sorafenib experienced, **B** REFLECT criteria, **C** HBV infection, **D** HCV infection

In L + P group, there was no significant difference regardless of sorafenib experienced and HCV infection (Fig. 3A, D). Patients with HBV infection (non-reached vs. 12.4 months, *p* = 0.16) and REFLECT criteria fit (non-reached vs. 14.6 months, *p* = 0.16) demonstrated a trend of better prognosis (Fig. 3B, C). For those patients who got response according to RECIST 1.1 criteria achieved longer tumor control and survival. The median OS were significantly different among patients with PR, SD, and PD (non-reached vs. 14.6 vs. 4.7 months, *p* = 0.03). The median PFS were 11.2 months, 6.4 months, and 2.2 months for patients with PR, SD, and PD respectively(*p* < 0.0001) (supplemental Fig. 1A, B).

### Prognostic factors

Among all the 87 HCC patients, HCV, PVT, and AFP > 400 ng/mL were poor prognostic factors and nivolumab used was protective factor in the univariate analysis. In multi-variates analysis, only PVT (HR 4.3, 95% C.I. 1.5–12.8) and AFP > 400 ng/mL (HR 3.3, 95% C.I. 1.1–9.3) were poor prognostic factors and nivolumab used remained a protective factor (HR 0.2, 95% C.I. 0.1–0.7) (Table 3).

**Table 3:** Prognostic factors for death (n = 87)

**Variables**	**Univariate HR (95% CI)**		***p* value**	**Multi-variates HR (95% CI)**		***p* value**
General						
Age ≥ 60	1.1	(0.5–2.8)	0.79			
Male	0.7	(0.3–1.7)	0.40			
HBV	0.6	(0.2–1.5)	0.27			
HCV	3.3	(0.9–13.0)	0.08	2.3	(0.5–10.0)	0.26
CPS class B	2.1	(0.8–5.5)	0.13			
Nivolumab used	0.4	(0.2–1.0)	0.05	0.2	(0.1–0.7)	0.01
HCC status						
PVT	2.9	(1.1–7.3)	0.03	4.3	(1.5–12.8)	0.01
Metastasis	1.0	(0.4–2.6)	0.94			
AFP > 400 (ng/mL)	2.6	(1.1–6.4)	0.03	3.3	(1.1–9.3)	0.03

*CPS* Child–Pugh score, *PVT* portal vein thrombosis, *AFP* alpha- fetoprotein

### Toxicity

In all grades of toxicities, dermatitis (35.0%), pruritis (27.5%), and hypothyroidism (27.5%) were most common. Few patients developed grade 3/4 toxicities including dermatitis (15.0%), GI bleeding (7.5%), hypertension (5.0%), pneumonitis (2.5%) and stomatitis (2.5%). Notably, only 5.0% of patients developed grade 1/2 hand-foot skin reaction. Severe adverse events were noted in 10% of patients, that included 2 gastric intestinal bleeding, 1 duodenal perforation and 1 pneumonitis (Table 4).

**Table 4:** Adverse events of the lenvatinib combined nivolumab group

**Adverse events**	**Grade 1/2**	**Grade 3/4**	**All grades**
Dermatitis	20.0%	15.0%	35.0%
Pruritus	27.5%	0.0%	27.5%
Fatigue	20.0%	0.0%	20.0%
Hypertension	15.0%	5.0%	20.0%
Diarrhea	17.5%	0.0%	17.5%
Dysphonia	12.5%	0.0%	12.5%
Stomatitis	10.0%	2.5%	12.5%
GI bleeding	0.0%	7.5%	7.5%
Pneumonitis	5.0%	2.5%	2.5%
HRSR	5.0%	0.0%	5.0%
Laboratory test			
Hypothyroidism	27.5%	0.0%	27.5%
Proteinuria	20.0%	0.0%	20.0%
Neutropenia	17.5%	0.0%	17.5%
Thrombocytopenia	15.0%	0.0%	15.0%
Anemia	10.0%	0.0%	10.0%
SAE*	0.0%	10.0%	10.0%

*HRSR* hand-foot skin reaction, *SAE* severe adverse event

^*^2 gastric intestinal bleeding, 1 duodenal perforation, 1 pneumonitis

## Discussion

To our knowledge, this is the first real-world report regarding the combination use of lenvatinib and nivolumab in advanced HCC which showed promising results with an ORR of 45.0% by mRECIST, PFS of 7.5 months, and OS of 14.6 months. These data suggested that lenvatinib plus nivolumab a potential combination in advanced HCC.

Cumulative evidence disclosed that the activation of FGF pathway signaling had an essential role in developing and worsening HCC [18]. Matsuki et al. performed in vitro studies in human HCC cell lines and in vivo studies in mice xenograft models showing that FGF19 –FGFR4 axis enhanced HCC proliferation and growth [19]. These findings may explain the high response rate of lenvatinib, a FGFR 1–4 inhibitor, in advanced HCC [4].

Lenvatinib, like other multiple kinase inhibitors, has been found to have immunomodulatory effects [14]. VEGFA and bFGF significantly upregulated the expression of immune-checkpoint markers and inhibited secretion of IFN-γ and granzyme B, which suppressed T cell cytotoxicity. This immunosuppressive effect was reverted by lenvatinib [20]. Another study showed the activation of FGFR signaling downregulated JAK/STAT pathway leading to the decrease of IFN-γ secretion. With the use of lenvatinib inhibited FGFR signaling restoring the IFN-γ stimulation [21]. In addition, several studies demonstrated that lenvatinib increased the percentage of activated CD8 + T cells that secreting IFN-γ and granzyme B [15, 21, 22]. However, the antitumor activity of lenvatinib was attenuated in immunodeficient mice by CD8 + T cell depletion [15]. On the other hand, lenvatinib decreased the proportion of monocytes and macrophages population, and tumor-associated macrophages (TAMs) [15, 21, 22]. Taking together, lenvatinib promoted anti-tumor immunity by increased IFN-γ–producing CD8 T-cell and decreased TAMs, which makes lenvatinib potential to combine with immunotherapy.

Lenvatinib combined with anti-PD-1 induced greater antitumor activity and had longer survival in animal model of renal cell carcinoma [21]. Two HCC syngeneic mouse model showed that the combination therapy increased more percentage of IFN-γ + and granzyme B + CD8 + T cells and decreased the macrophages population [15, 22]. Combined therapy also reducedPD-1 + T cells and modulated inflammatory factors which had an extensive immunomodulatory effect in the tumor microenvironment in HCC mouse model. Additionally, the immunomodulatory effect was more potent when combined with lenvatinib than sorafenib [20].Therefore, the combination of lenvatinib and anti-PD-1 synergistically modulated the TME and enhanced antitumor immunity.

Lenvatinib combined anti-PD-1 has been approved in endometrial carcinoma and renal cell carcinoma. In KEYNOTE-775/Study 309, lenvatinib and pembrolizumab demonstrated an ORR of 30%, PFS 6.6 months (HR 0.60) and OS 17.4 months (HR 0.68), which was superior to doxorubicin or paclitaxel in platinum-experienced endometrial carcinoma [23]. In renal cell carcinoma, the combination of lenvatinib and pembrolizumab was highly effective with an ORR of 38% in the phase Ib/II trial KEYNOTE-146 [24]. In the phase III CLEAR trial, lenvatinib combined with pembrolizumab showed a longer PFS (23.9 vs. 9.2 months; HR 0.39) and OS (HR 0.66) than sunitinib [25]. In HCC, a phase Ib study of lenvatinib plus nivolumab showed the ORR of 54% and PFS of 7.4 months [16]. Another phase I-II study of lenvatinib plus pembrolizumab had the ORR of 36% and PFS of 8.6 months [26]. These clinical trials revealed promising results that is comparable with the synergistic effect in animal model.

For the treatment outcomes of other combined regimens in advanced HCC, a global open-labeled phase III trial (IMbrave150) revealed atezolizumab plus bevacizumab had a higher ORR (27.3% vs. 11.9%), longer PFS (6.8 vs. 4.3 months) and OS (non-reached vs. 13.2 months) then sorafenib [8]. In the phase II study of CheckMate-040, the combination nivolumab and ipilimumab yielded an ORR of 27–32% andOS (12.5–22.8 months) among different dose of combination in HCC patients who progressed from sorafenib [27]. In our study, lenvatinib combined nivolumab showed an objective response rate of 45% and a median PFS of 7.3 months. Therefore, our study demonstrated comparable efficacy to the treatments mentioned above.

The most adverse effects were graded 1–2 in this study. Skin reactions such as dermatitis and pruritis, and hypothyroidism were most common and could be caused by lenvatinib or nivolumab. Severe AEs including two gastric intestinal bleeding and one duodenal perforation were more likely related to lenvatinib; one patient suffered grade 5 pneumonitis was likely related to nivolumab. Overall, the toxicity profile was comparable to those reported in the phase Ib study of lenvatinib combined nivolumab and phase I-II study of lenvatinib combined pembrolizumab [16, 28].

There were several limitations in our study. First, this is a retrospective study, the information bias and selection bias may exist. However, imbalanced factors of age and HBV infection were not independent risk factors. Therefore, this study still provided a proof of concept. Second, whether the results apply to non-Asian populations is unclear because of the different etiologies between Asian and Western countries. Nevertheless, the response and prognosis were not significantly different regardless of virus infection.

## Conclusion

This study is by far the first real-world data regarding the combination of lenvatinib and nivolumab in advanced HCC. We reported the promising efficacy and tolerable toxicities that is comparable with the clinical trial. Whether this regimen can become standard of care remains to be confirmed in a large prospective clinical trial.

## Supplementary Information

Below is the link to the electronic supplementary material.

Supplementary file1 Fig. 1. Kaplan–Meier curves for (A) progression-free survival and (B) overall survival stratified by treatment response (RECIST criteria) (TIF 4687 KB)

Supplementary file2 Fig. 2. Case with complete response by mRECSIT criteria Ill-defined mass lesion at posterior segment of liver (arrow) and portal vein thrombus (dotted arrow).Two months later, the tumor was regressed. Six months later, both tumor and portal vein thrombus achieved complete response. In the meanwhile, his tumor marker declined to normal range (TIF 3001 KB)

## Data Availability

Data available on request from the authors.
