# Supplementary figures and images for: Lenvatinib combined with nivolumab in advanced hepatocellular carcinoma-real-world experience
Source: Invest New Drugs. 2022 Apr 28;40(4):789–97. doi: 10.1007/s10637-022-01248-0 (PMC9288359; doi:10.1007/s10637-022-01248-0)

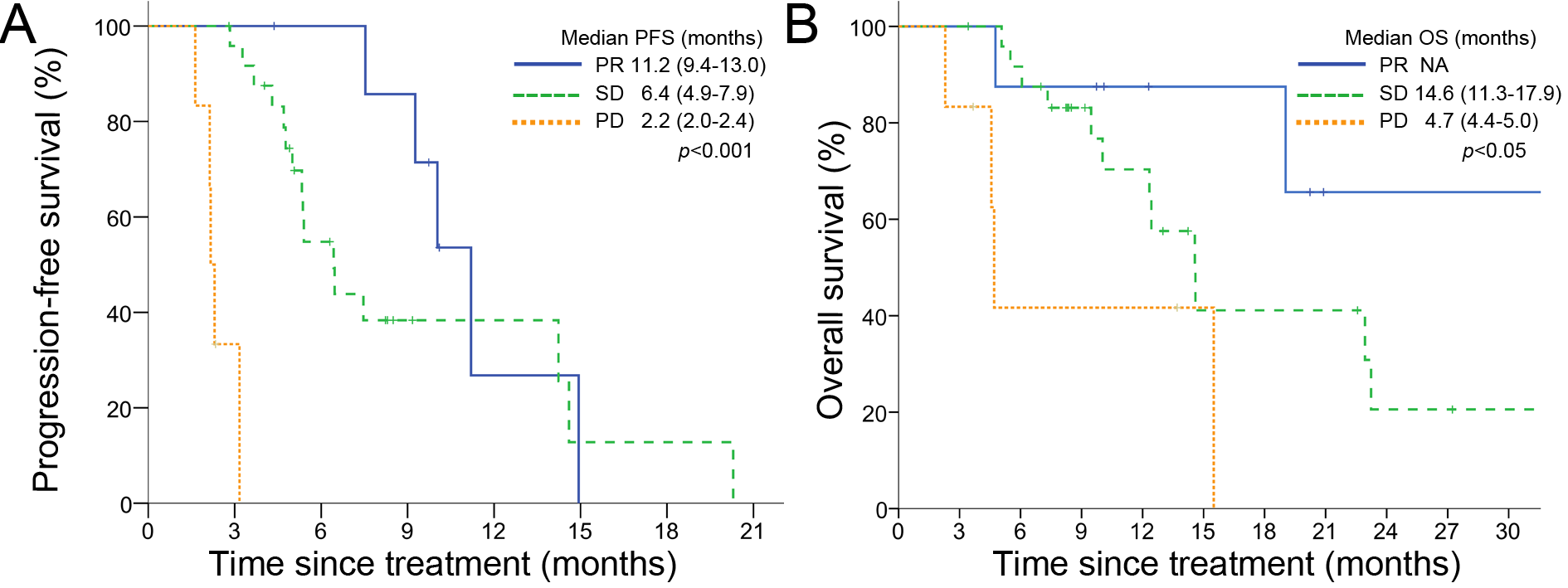

Supplement: Supplementary file 1 — Supplementary file1 Fig. 1. Kaplan–Meier curves for (A) progression-free survival and (B) overall survival stratified by treatment response (RECIST criteria) (TIF 4687 KB) [file 10637_2022_1248_MOESM1_ESM.tif]

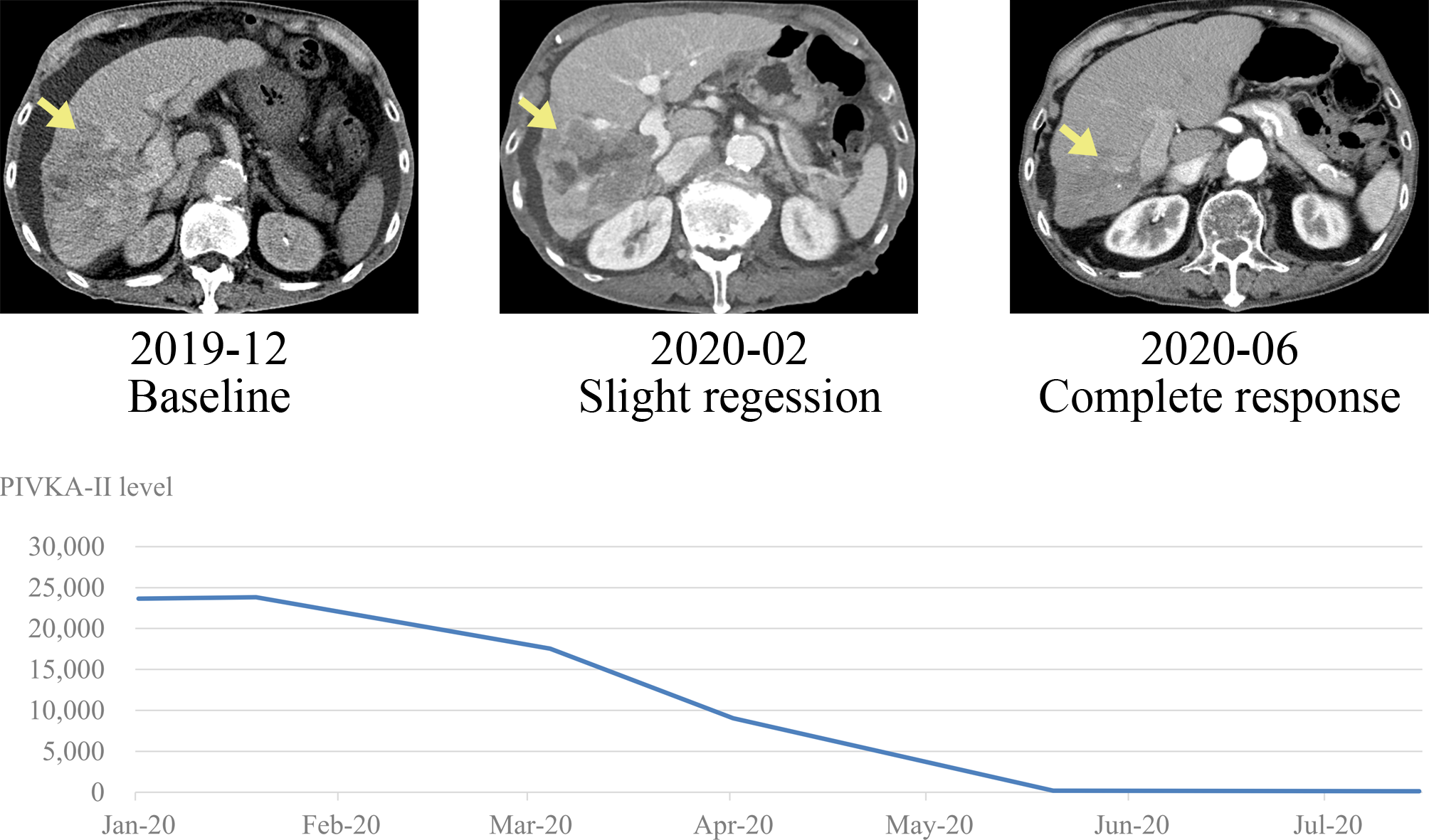

Supplement: Supplementary file 2 — Supplementary file2 Fig. 2. Case with complete response by mRECSIT criteria Ill-defined mass lesion at posterior segment of liver (arrow) and portal vein thrombus (dotted arrow).Two months later, the tumor was regressed. Six months later, both tumor and portal vein thrombus achieved complete response. In the meanwhile, his tumor marker declined to normal range (TIF 3001 KB) [file 10637_2022_1248_MOESM2_ESM.tif]
